# Sport expertise and visuomotor performance: Evidence from 598 athletes aged 14-39 years

**DOI:** 10.1080/11791543.2026.2630457

**Published:** 2026-02-20

**Authors:** Viktor Veselý, Pavel Kolář, Martin Macaš, Jáchym Kolář, Alena Kobesová

**Affiliations:** a Department of Rehabilitation and Sports Medicine, Second Faculty of Medicine, Charles University and University Hospital Motol, Prague, Czech Republic; b Czech Institute of Informatics, Robotics and Cybernetics, Czech Technical University in Prague, Prague, Czech Republic

**Keywords:** Elite athletes, sports vision, perceptual-motor performance, visual-motor skills, multiple object tracking, near-far quickness

## Abstract

Visuomotor functions are critical to athletes’ perception, decision-making, and motor execution. This retrospective cross-sectional study analyzed 598 athletes (365 male, 233 female; 14–39 years) assessed with a unique combination of standard optometric tests and a digital battery across 10 visuomotor domains. Participants were categorized by competitive level (professional/non-professional) and sport type (strategic/interceptive). A semiparametric Bayesian model examined associations between athlete characteristics and visuomotor scores, and a self-organizing map visualized multivariate profiles. Professionals showed credible advantages in six of ten domains, including depth perception, perception span, reaction time, multiple object tracking, and near-far quickness by both methods. Visual clarity and contrast sensitivity did not differ, and binocular vergence facility and binocular accommodation facility showed small, non-credible differences. Female athletes showed better depth perception and binocular accommodation facility, whereas male athletes performed better in contrast sensitivity and multiple object tracking. Strategic sport athletes outperformed interceptive athletes in multiple object tracking. Multivariate results showed close links between perception span and multiple object tracking and between binocular vergence and binocular accommodation facilities. These findings indicate that higher competitive status is associated with superior dynamic and cognitively demanding visuomotor functions, although the cross-sectional design precludes causal inference. The results support targeted visuomotor profiling in athletes.

## 1 Introduction

Visual perception plays a fundamental role in human motor behaviour, particularly in sports, where athletes rely on rapid integration of visual information to guide decisions and execute precise motor actions. On average, individuals perform over 250,000 eye movements daily, highlighting the extensive involvement of the visual system in everyday functioning (Oh et al., 2018). In sports, visual input is integrated with other sensory cues to regulate motor responses, forming what are referred to as visuomotor or visual-motor functions (VMF).

Athletes with superior VMF often demonstrate better decision-making, anticipation, and motor execution. Meta-analyses indicate that elite athletes generally outperform lower-level athletes on perceptual-cognitive tasks such as anticipation and attentional processing (Mann et al., 2007; Voss et al., 2010). Electrophysiological studies suggest that elite athletes’ enhanced visuomotor reaction times may reflect superior visual processing rather than motor execution alone (Hülsdünker et al., 2018). Similar work using objective neurophysiological indices in elite athletes has underscored the value of instrumented, performance-linked cognitive assessment in this population (Kirby et al., 2025).

VMF abilities may also vary by sport type, reflecting sport-specific perceptual and cognitive demands. Interceptive sports (e.g., tennis, fencing) require accurate coordination between body and object, while strategic sports (e.g., football, basketball) emphasise rapid integration of complex spatial information and tactical decision-making (Burris et al., 2020).

Recent findings suggest that VMF are not solely innate but can improve through training. Specific visual functions have been linked to in-game performance statistics (Poltavski & Biberdorf, 2015) and vision training and neurofeedback-based interventions have enhanced dynamic acuity, anticipation, decision-making, and even injury prevention (Clark et al., 2020; Romeas et al., 2016; Yu et al., 2025; Zwierko et al., 2015). Related sports-medicine work has also shown that visual-cognitive measures are sensitive to sport-related brain stress such as concussion, further supporting their clinical relevance (Covassin et al., May 2010).

However, no standardised testing battery exists for VMF testing. Tools like the Nike SPARQ Sensory Station and its successor, the Senaptec Sensory Tablet, have been used to evaluate multiple visual-perceptual domains (Wang et al., 2015), and brief vision-based sideline tests such as the King-Devick have also shown clinical utility in sport settings, underscoring the relevance of oculomotor/visuomotor screening in athletes (Legarreta et al., 2019).

This study aimed to compare VMF profiles between professional and non-professional athletes and to examine whether sport type and sex influence these profiles. A large cohort of athletes was assessed using both clinical optometry and digital VMF tests to evaluate differences related to competitive level, sport-specific demands, and biological sex. Given the retrospective cross-sectional design, observed group differences are interpreted as associations rather than direct proofs of causation. To reduce developmental and aging confounds, an age-restricted athletic sample (14-39 years) was analysed (Allen & Hopkins, 2015; Niechwiej-Szwedo et al., 2020). A semiparametric Bayesian copula and Self-Organising Map visualisation were used to characterise conditional dependencies and multivariate profiles across VMF domains - going beyond univariate contrasts to provide translational context for test selection, screening, and sport-specific training (Hoff, 2007; Sarvestan et al., 2023). Beyond these aims, the study advances prior work by combining a validated digital battery (SST) with clinical optometry measures within the same cohort, enabling convergent assessment of overlapping constructs (e.g., near-far quickness from both tablet and optometric protocols) (Burris et al., 2020; Erickson et al., 2011; Wang et al., 2015).

## 2 Materials and methods

### 2.1 Participants

This retrospective, cross-sectional study analysed visuomotor data from routine assessments at a sports vision and optometry centre. This study was approved by the Ethics Committee of the University Hospital Motol (Reference No. EK-328/25) and all procedures adhered to the Declaration of Helsinki. Because the analysis used retrospectively collected, fully anonymized clinical data, the ethics committee waived the need for individual informed consent.

Anonymized raw data were obtained from a single sports optometry centre, with consent secured by the study author's team. The dataset included demographic information and VMF parameters, including Senaptec Sensory Tablet (SST) results. Initially, 1,964 examinations from 788 individuals were collected, with some undergoing repeated assessments. To ensure each participant was represented only once, only the first examination per subject was included in the analysis.

Participants were categorised by self-reported primary sport and grouped according to sport-specific demands. Since no universally accepted taxonomy exists, we adopted a widely used approach from recent literature (Burris et al., 2020; Voss et al., 2010; Yu & Liu, 2020), dividing sports into Strategic, Interceptive, Static, and Other categories. Strategic sports involve processing rapidly changing information, such as player positions and ball movement (e.g., football, basketball). Interceptive sports require coordination between the body, held tools, and environmental objects (e.g., tennis, fencing) (Burris et al., 2020; Voss et al., 2010; Yu & Liu, 2020). Static sports are characterised by consistent, self-paced actions (e.g., long-distance running, swimming) (Voss et al., 2010). Other sports include unique activities that do not fit the previous categories (e.g., sledding, dance). Due to the low number of participants in the Static and Other categories and the unclear role of VMF in these sports, these groups were excluded from the analysis.

Participants under 14 were excluded due to ongoing development of visuomotor functions and because 14 is generally the minimum age for professional athletic status (Burris et al., 2020; Niechwiej-Szwedo et al., 2020). Participants over 39 years of age were also excluded, as elite athletic performance typically peaks before 40 and visual functions show a gradual decline from this age onward (Allen & Hopkins, 2015).

Optometrists from the sports optometry centre were consulted on their criteria for classifying sport levels during SST testing. Standardised clinic intake forms and an optometrist interview at testing were used to assign competitive status, which reflected the athlete’s reported training frequency and competition level at the time of testing. Athletes training at least twice weekly and competing nationally or internationally were labelled Professional/Elite; others were classified as Recreational, Middle School, High School, or College, based on training frequency, competition level, and age. For the primary analyses, competitive status was dichotomised as Professionals (Professional/Elite: ≥ 2 training sessions per week and national/international competition; hereafter “Pro”) versus Non-Professionals (all remaining above-mentioned categories not meeting the Professional/Elite criteria; hereafter “NoPro”). Participants labelled as Beginner, Other, Retired, or Youth were excluded due to insufficient sport-level data.

**Figure 1.**
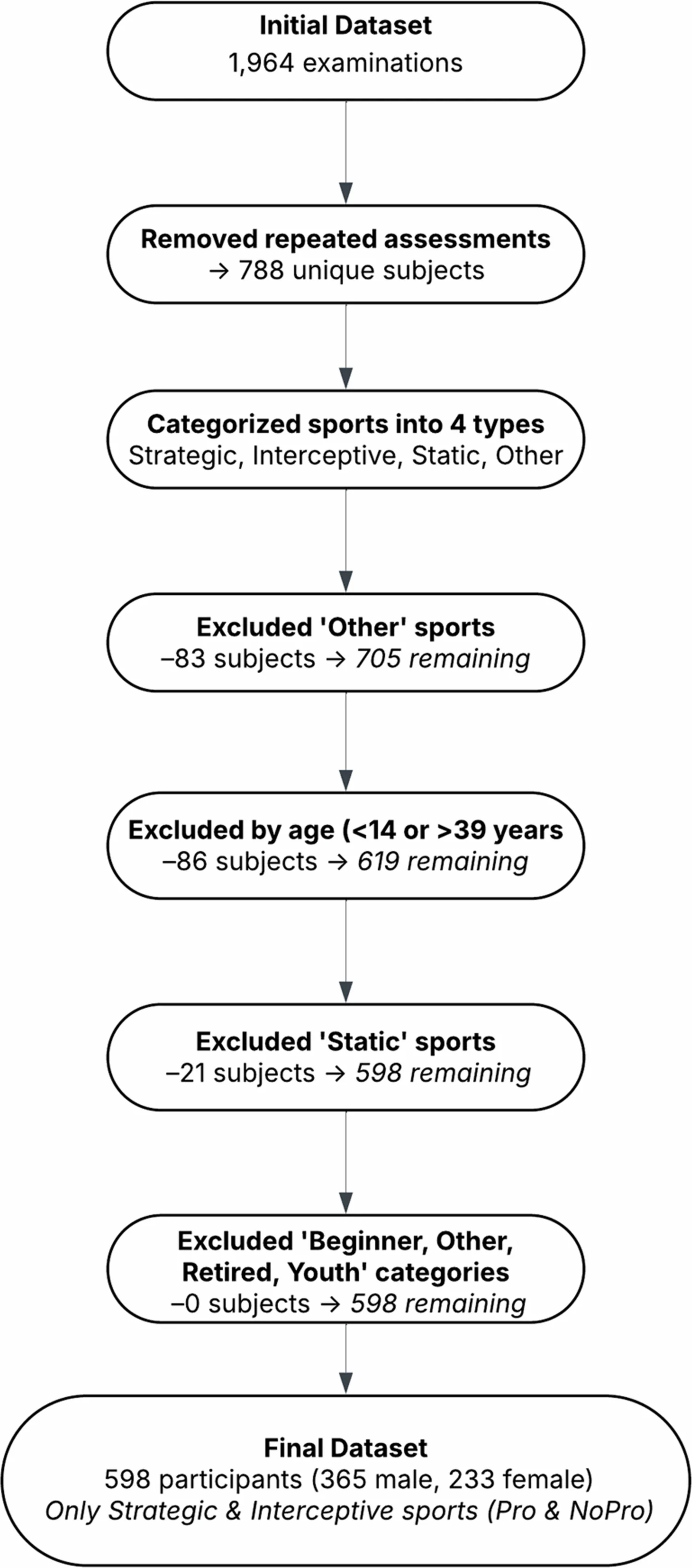
Flowchart of participant selection for data analysis (*Pro = Professional Athletes, NoPro = Non-Professional Athletes).*

After applying all criteria, 598 athletes (365 male, 233 female; aged 14-39) were included. Figure 1 shows the selection flow. No structured sports vision/visuomotor training was reported among participants, according to optometrist interviews; however, training exposure was not captured in a standardised quantitative form (e.g., weekly training volume, years in sport, prior vision training/therapy), and unmeasured exposure cannot be ruled out.

### 2.2 Design

This retrospective, cross-sectional, observational study assessed VMF under real-world clinical conditions without experimental manipulation. The goal was to examine associations between VMF performance and athlete characteristics (professional status, sport type, sex).

### 2.3 Methodology

Personal history included age, sex (see Table 1), primary sport, performance level, position, limb and eye dominance, concussion, eye trauma/surgery, and eye care history. Tests were conducted with the participant’s habitual correction (i.e., with glasses or contact lenses, depending on what they typically use during sports). All assessments were administered by trained optometrists, at eye level under standard clinic lighting per device and test instructions.

**Table 1.** Summary table of participant characteristics.

Distribution of age among athlete groups (age in years)							
Female athletes				Male athletes			
Sport level	NoPro	Pro	Total		NoPro	Pro	Total
Strategic [years]	19.60 ± 3.69	18.51 ± 3.89	18.65 ± 3.87	Strategic	18.85 ± 4.54	20.86 ± 6.16	20.47 ± 5.94
Interceptive [years]	23.57 ± 5.81	21.44 ± 5.17	21.82 ± 5.32	Interceptive	26.00 ± 6.63	23.06 ± 5.74	23.38 ± 5.85
Total [years]	21.24 ± 5.01	19.45 ± 4.54	19.71 ± 4.65	Total	19.51 ± 5.15	21.22 ± 6.14	20.91 ± 6.01

Distribution of athlete level and sport type (number)							
Female athletes				Male athletes			
Sport level	NoPro	Pro	Total		NoPro	Pro	Total
Strategic	59	251	310	Strategic	20	135	155
Interceptive	6	49	55	Interceptive	14	64	78
Total	65	300	365	Total	34	199	233

Summary table of participant characteristics. In the upper part the distribution of age in years among athlete groups represented as mean ± standard deviation and in the lower part distribution of athlete level and sport type as a number of participants in each group. Pro category includes athletes from Professional/Elite athletes’ group and NoPro category includes athletes from Recreational, Middle School, High School, and College groups. Age distributions by subgroup are provided for transparency; models did not adjust for age.

Near-Far Quickness (NFQO) was assessed using a modified Haynes Distance Rock Test (Haynes, 1979) with charts at 40 cm and 6 m distance from the participant’s eyes.

Binocular Accommodation Facility (BAF) was assessed using the Accommodative Lens Rock Test with ± 2D flipper lenses and an Accommodative Rock Card (ARC) at 40 cm (Bollano-Lazaridis & Chandrinos, 2021; Wick et al., 2002).

Binocular Vergence Facility (BVF) was assessed using the same protocol as BAF but with prismatic flipper lenses 3ΔBI/12ΔBO prismatic dioptres.

The Senaptec Sensory Tablet (SST) (M&S Technologies, Inc., Niles, IL) is a 13.3" touchscreen device used to assess and train visuomotor abilities. Adjusted to the participant’s eye level, it connects via Bluetooth to a smartphone, serving as the input device. Its test-retest reliability and construct validity have been documented (Erickson et al., 2011; Wang et al., 2015). The SST evaluates seven visuomotor abilities: Visual Clarity (VC), Contrast Sensitivity (CS), Depth Perception (DP), Near-Far Quickness (NFQS), Perception Span (PS), Reaction Time (RT), and Multiple Object Tracking (MOT). For brief descriptions, see Table 2; detailed task descriptions are available in Kramer et al. (2021) (Kramer et al., 2021).

**Table 2.** Summary of tested Visuomotor functions.

Task (Measurement method^1^)	Abbreviation	Units	Task Description
Visual Clarity (SST)	VC	logMAR^2^	Visual acuity at a 3m distance
Contrast Sensitivity (SST)	CS	logCS^3^	The minimum perceived difference in contrast at a 3m distance
Depth Perception (SST)	DP	Arcsecond^4^	The ability to estimate depth differences at a 3m distance
Perception Span (SST)	PS	Number of correct responses	The ability to remember and recall visual patterns
Reaction Time (SST)	RT	milliseconds	The ability to quickly react to a simple visual stimulus
Multiple Object Tracking (SST)	MOT	Score	The ability to divide attention by identifying target circles after they move among identical non-target circles
Near-Far Quickness (SST)	NFQS	Number of correct responses	The number of characters correctly reported within 30 seconds when alternating near (hand distance) and far distance (3m) characters
Binocular Vergence Facility (Optometrist)	BVF	Cycles per minute (cpm)	Number of cycles per minute (two flipper rotations) while reading ARC at 40 cm through 3ΔBI/12ΔBO prismatic lenses.
Binocular Accommodation Facility (Optometrist)	BAF	Cycles per minute (cpm)	Number of cycles (2 rotations of flipper) per minute while reading ARC at 40 cm distance through flipper with ± 2D lenses
Near-Far Quickness (Optometrist)	NFQO	Seconds (s)	Time to correctly read ten character pairs while alternating between near (40 cm) and far (6 m) distances.

^1^ Measurement method indicates whether the method was tested by the optometrist (Optometrist) or measured automatically by Senaptec Sensory Tablet (SST).

^2^ logMAR is a logarithm of Minimal Angle of Resolution assessed on optotypes such as Snellen Chart, score 20/20 (normal visual acuity) on Snellen Chart equals 0 in log(MAR), negative results in log(MAR) represents better than normal visual acuity and positive results represents worse than normal visual acuity.

^3^ logCS logarithm of reached threshold for 6 resp. 18 cycles per degree frequency.

^4^ An arcsecond is an angular measurement equal to 1/3600 of a degree.

### 2.4 Statistical analysis

To examine the relationships between VMF parameters and athlete attributes, we employed semiparametric Bayesian Gaussian copula estimation (Hoff, 2007). This method was chosen for its ability to model non-normal and differently scaled distributions, handle binary covariates (sex, sport type, and professional status) jointly with continuous outcomes, and estimate conditional multivariate dependencies among visual task scores within a single framework. This allowed us to analyse all VMF parameters together, estimate associations with athlete attributes, and map task-task dependencies in a coherent model. To ensure consistent interpretation, all VMF parameters were adjusted so that higher values uniformly indicate better performance. For measures where lower values originally reflected better performance, specifically VC, NFQO, RT, and DP, the scales were reversed prior to analysis.

The Bayesian model was implemented using Gibbs sampling, with 30,000 iterations, retaining every 10^th^ scan to mitigate autocorrelation. The first 1,000 samples were discarded as burn-in. Model convergence was assessed using the Gelman-Rubin statistic (PSRF), with values between 0.9998 and 1.0046, confirming stable posterior estimates (Gelman & Rubin, 1992). Trace plots further validated convergence by demonstrating proper chain mixing (Roy, 2020). Posterior estimates for the regression coefficients, along with 95% credible intervals, were computed to assess associations between athlete attributes and VMF parameters. A relationship was considered statistically credible when the 95% credible interval excluded zero (i.e., at least 95% of the posterior distribution lay on one side of zero). In Bayesian terms, “statistically credible” indicates that the data provide strong posterior evidence for an effect, analogous to statistical significance in frequentist inference, but based on probability distributions rather than hypothesis testing. Within the restricted age range (14-39 years), models did not adjust for age or training exposure due to lack of standardised training-dose data (e.g., weekly hours, years in sport).

To explore multivariate relationships and cluster structures in the dataset, a Self-Organising Map (SOM) neural network was trained using the SOM Toolbox in MATLAB (Sarvestan et al., 2023). The SOM projected the multidimensional visual score data onto a 15 × 7 toroidal grid, preserving topological relationships and facilitating visualisation of both group clustering and interdependencies among visual functions.

Given the retrospective and exploratory nature of the study, no a priori sample size calculation was conducted. However, the final sample of 598 participants exceeds those of most comparable studies in this area (Murray et al., 2021; Poltavski & Biberdorf, 2015; Zwierko et al., 2015), supporting the statistical robustness and generalisability of the findings.

## 3 Results

**Figure 2.**
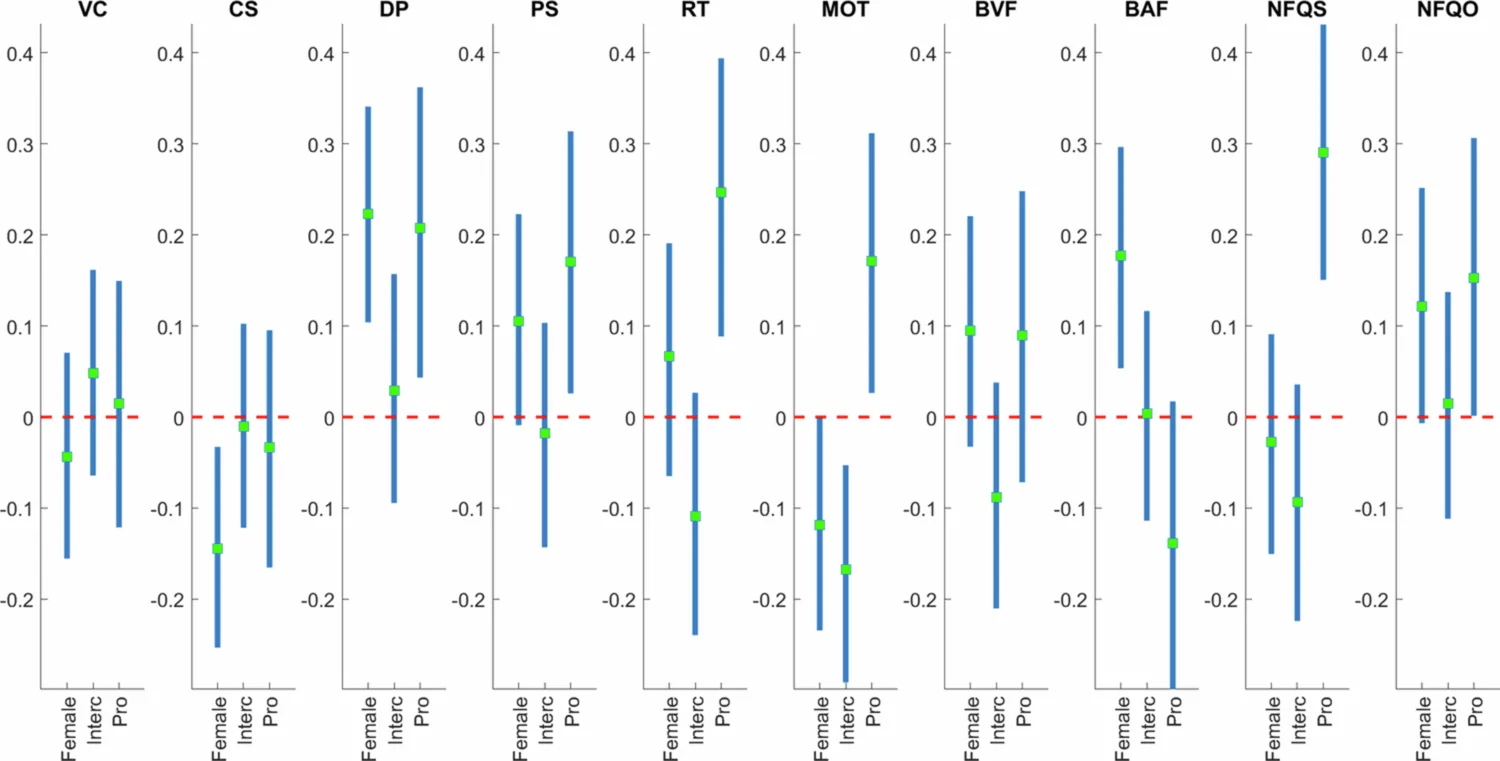
Posterior means of regression coefficients with 95% credible intervals (CIs). The red line represents the baseline, corresponding to the reference group (NoPro male athlete in Strategic sports). Positive coefficients reflect better visuomotor performance relative to this reference group. All visual scores were transformed prior to analysis so that higher values consistently indicate better performance across all variables. Credible intervals that do not cross zero indicate statistically credible differences.

Bayesian analysis revealed statistically credible associations between athlete attributes and visuomotor performance (Figure 2). Pro athletes showed statistically credible higher scores (i.e., better) scores in Depth Perception (DP), Near-Far Quickness (NFQS, NFQO), Perception Span (PS), Reaction Time (RT), and Multiple Object Tracking (MOT), indicating superior VMF in these domains. No statistically credible differences were observed between professional and non-professional athletes in Visual Clarity (VC) or Contrast Sensitivity (CS). For Binocular Vergence Facility (BVF) and Binocular Accommodation Facility (BAF), posterior medians suggested small differences by status, but the 95% credible intervals included zero and were therefore not statistically credible.

Sex differences were observed, with female athletes showing higher DP and BAF scores, but lower CS and MOT scores compared to males. Sport type comparisons indicated that Strategic sport athletes outperformed Interceptive sport athletes in MOT, suggesting enhanced ability to track moving objects in dynamic environments.

**Figure 3.**
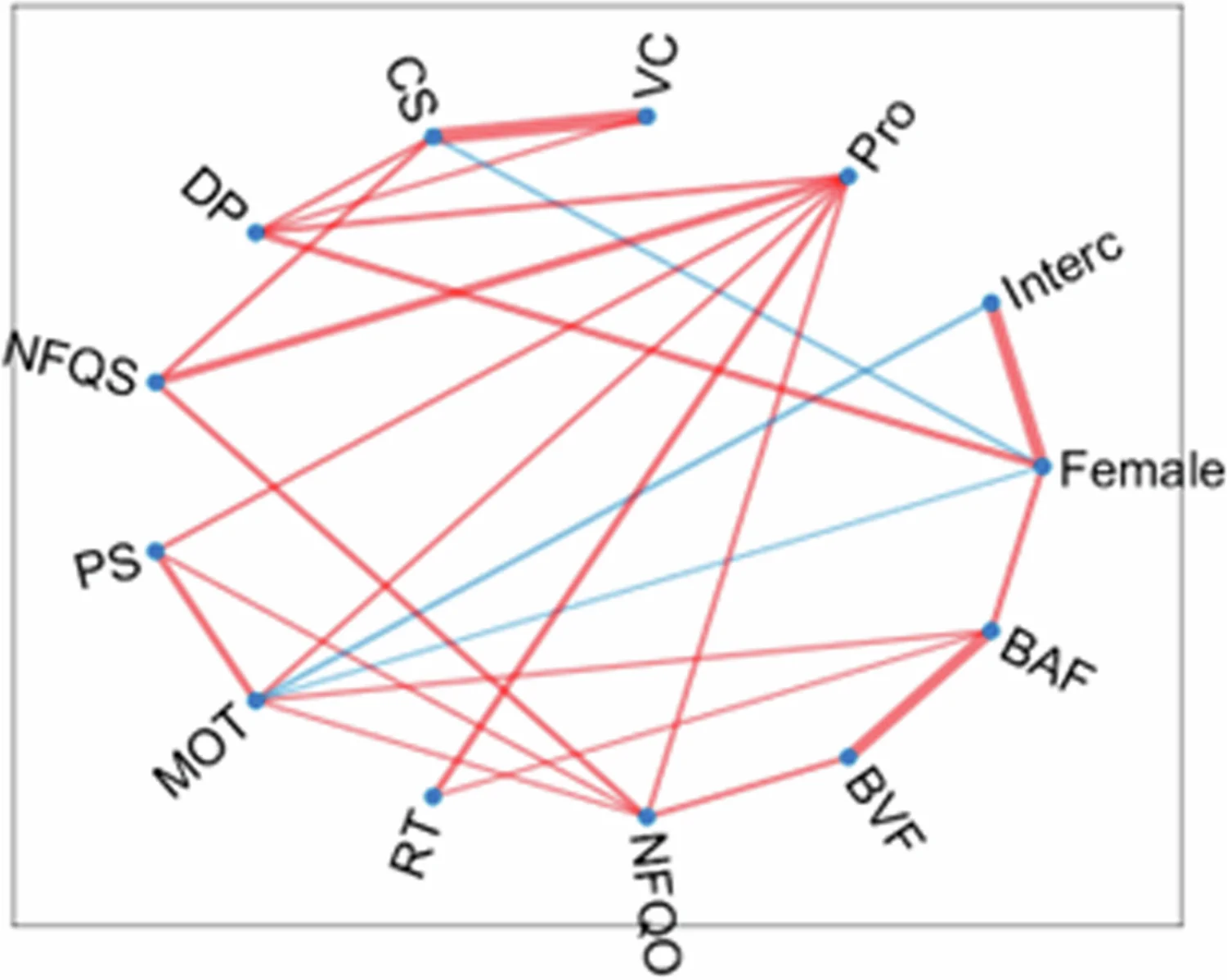
Conditional dependence graph of athlete attributes and visuomotor parameters. Edges between nodes indicate relationships where the 95% credible interval (CI) does not include zero. Red edges denote positive associations, while blue edges indicate negative associations. The width of each edge reflects the relative strength of the conditional relationship (thicker edges represent stronger statistical associations between the variables).

The conditional dependence graph (Figure 3) further revealed several strong multivariate relationships. The strongest associations were observed between CS and VC, and between PS and MOT, both demonstrating particularly strong conditional dependencies. Additional robust associations were identified between BAF and BVF. Furthermore, a statistically credible association was observed between Interceptive sport type and female sex, reflecting the sport-type composition of the sample. This composition cautions against over-attributing sex effects to biology alone.

Statistical credibility for all associations was determined using 95% credible intervals (CIs); relationships were considered credible when the CI excluded zero, indicating strong Bayesian support for the effect.

Table 3 summarises the posterior estimates for each athlete attribute across all VMF (i.e., the most probable values of the parameters after taking the data into account). Notably, the Professional status (Pro) variable showed the most consistent positive associations across multiple VMF, reinforcing the importance of VMF in elite athletic performance.

**Table 3.** Posterior percentiles of regressioncoefficients for athlete attributes across visuomotor parameters.

	VC			CS			DP			PS			RT		
	Fem	Inter	Pro	Fem	Inter	Pro	Fem	Inter	Pro	Fem	Inter	Pro	Fem	Inter	Pro
**P5**	−0.155	−0.064	−0.121	−**0.253**	−0.122	−0.165	**0.104**	−0.094	**0.043**	−0.009	−0.143	**0.026**	−0.065	−0.239	**0.088**
**P50**	−0.044	0.048	0.015	−**0.144**	−0.010	−0.033	**0.223**	0.029	**0.208**	0.105	−0.017	**0.171**	0.067	−0.109	**0.247**
**P95**	0.070	0.162	0.149	−**0.033**	0.102	0.095	**0.341**	0.157	**0.362**	0.223	0.103	**0.314**	0.191	0.027	**0.394**

	MOT			BVF			BAF			NFQS			NFQO		
	Fem	Inter	Pro	Fem	Inter	Pro	Fem	Inter	Pro	Fem	Inter	Pro	Fem	Inter	Pro
**P5**	−0.234	−**0.291**	**0.026**	−0.033	−0.210	−0.072	**0.054**	−0.114	−0.299	−0.150	−0.224	**0.151**	−0.007	−0.112	**0.001**
**P50**	−0.119	−**0.168**	**0.171**	0.095	−0.089	0.089	**0.177**	0.003	−0.138	−0.028	−0.093	**0.291**	0.121	0.015	**0.153**
**P95**	0.001	−**0.053**	**0.312**	0.221	0.038	0.248	**0.297**	0.116	0.017	0.091	0.036	**0.431**	0.252	0.137	**0.306**

The 5th (P5), 50th (P50), and 95th (P95) percentiles represent the posterior distribution of regression coefficients, indicating the strength and direction of associations between binary athlete attributes (sex, sport type, professional status) and visuomotor parameters. Credible associations are identified where the 95% credible interval does not include zero, signifying statistically supported relationships. The statistically credible values are marked in **BOLD**.

**Figure 4.**
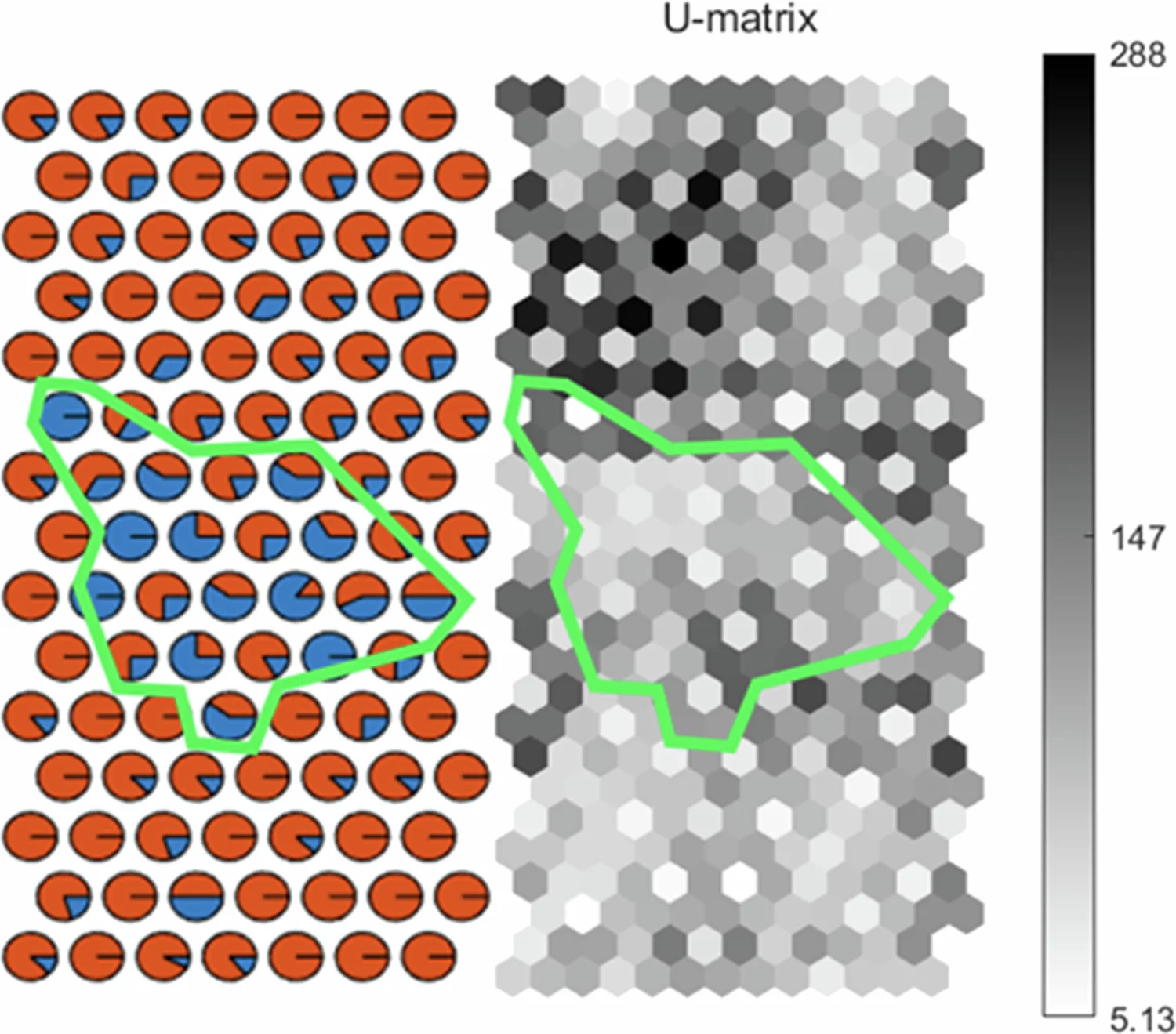
Self-Organising Map (SOM) representation of athlete distributions based on visuomotor parameters. The left panel shows pie charts representing the proportion of Pro (red) and NoPro (blue) athletes within each SOM unit. The green-outlined region highlights a cluster dominated by NoPro athletes. The right panel (U-Matrix) visualises distances between SOM units, with darker areas indicating stronger separations between clusters. The green-outlined region corresponds to a distinct grouping identified within the SOM space. These results highlight systematic differences in visuomotor profiles between Pro and NoPro athletes.

The Self-Organising Map (SOM) analysis further visualised multivariate performance profiles based on the full set of visual scores (Figure 4). Professional athletes clustered predominantly in the peripheral regions of the SOM grid, while non-professionals were concentrated in the central areas. This separation illustrates systematic differences in VMF profiles between the two groups. These visualisations corroborate the patterns observed in the Bayesian model. The unified distance matrix (U-matrix) confirmed distinct clustering patterns.

**Figure 5.**
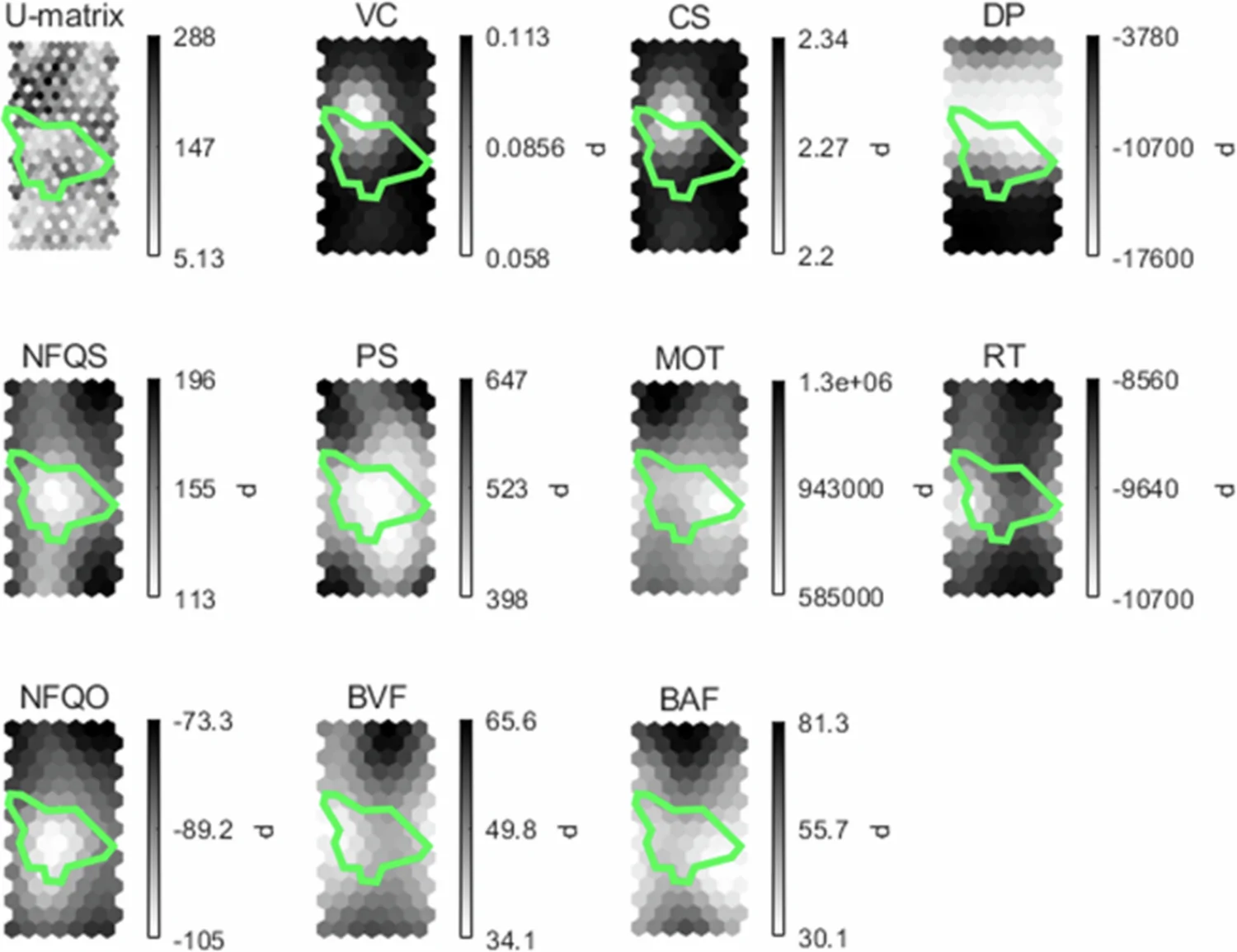
Self-Organising Map (SOM) heatmaps of visuomotor parameters. Each panel represents the spatial distribution of a specific visuomotor function (visuomotor functions’ parameter/score) across the SOM grid. Darker regions reflect higher visuomotor functions’ performance parameters, while lighter regions reflect lower parameters. For visual functions showing statistically credible differences (e.g., NFQO, NFQS, DP, PS, MOT, RT), higher-performing regions generally align with areas dominated by PRO athletes as seen in Figure 4. The green-outlined region highlights the NoPro-dominated cluster identified in the U-Matrix (top-left panel), showing systematic differences in visual task performance. The U-Matrix panel visualises distances between SOM units, with darker areas indicating stronger separations between clusters.

SOM heatmaps (Figure 5) showed that professional-dominated regions were aligned with higher performance in NFQS, NFQO, PS and MOT. MOT and RT also demonstrated lower (lighter) values in non-professional regions, consistent with Bayesian analysis. DP displayed a more mixed pattern, suggesting additional factors may contribute to its variability. The SOM visualisation also highlighted interdependencies among visual functions, with similar spatial distributions observed for VC-CS, NFQS-NFQO, and BVF-BAF-MOT, while DP and RT emerged as more independent dimensions.

## 4 Discussion

In this clinic-based cohort of 598 athletes aged 14−39, professional athletes outperformed non-professionals on several VMF parameters central to sport, namely in six out of ten VMF including Depth Perception (DP), Perception Span (PS), Reaction Time (RT), Multiple Object Tracking (MOT), and Near-Far Quickness (both NFQS and NFQO). No statistically credible differences were found in Visual Clarity (VC) and Contrast Sensitivity (CS), suggesting that these fundamental visual abilities could be less influenced by sports training. A trend toward superior Binocular Vergence Facility (BVF) but slightly worse Binocular Accommodation Facility (BAF) was observed in professional athletes; however, these differences were not statistically credible (95% credible intervals included zero) and should be interpreted cautiously. These findings suggest that sports expertise is associated with enhanced VMF, particularly in cognitive-perceptual tasks relevant to athletic performance.

The graded nature of the differences by competitive level and their sport-type specificity are what one would expect under a sustained, sport-specific training account and may also reflect selection. Accordingly, we interpret the pattern as consistent with training-related adaptation, while recognising alternative explanations. Because the design is retrospective and cross-sectional, we treat these relationships as associations rather than proofs of causation. Notably, effects were largest on tasks with higher cognitive-motor load, whereas low-level measures (e.g., simple acuity) showed smaller or absent differences, aligning with a training-demand interpretation. Relative to prior large datasets that relied solely on digital tasks, our integration of clinic-based optometry with SST provides a broader view of VMF, including two independent near-far quickness modalities. The age-restricted athletic cohort reduces developmental and aging confounds common in mixed-age samples, and the copula-based network reveals how VMF domains covary in athletes, information not captured by univariate contrasts alone. Together with SOM maps of multivariate profiles, these features add translational context for test selection, screening, and targeted training.

The lack of credible differences in VC and CS aligns with previous studies (Burris et al., 2020; Stalin & Dalton, 2020), which suggest that these basic visual skills are more likely to be influenced by anatomical and physiological factors rather than by training. In contrast, the superior performance of Pro athletes in more complex VMF tasks supports the notion that elite performance is driven by enhanced perceptual-motor processing rather than simple visual acuity. With respect to BVF and BAF, the present results do not provide statistically credible evidence of expertise-related differences, and their sport relevance should be interpreted cautiously. Facility tests performed with prism or lens flippers at a near testing distance impose optically induced dissociation demands that can partially uncouple vergence and accommodation, whereas in most sport viewing conditions these responses typically change together in response to real distance shifts and remain naturally coupled. In classic sports-vision battery work, athletes differed from nonathletes on several visual skills, but accommodative facility did not show a significant group difference (Christenson & Winkelstein, 1988). Omar et al. (2017) reported better vergence facility but worse accommodative facility in teenaged athletes; nonetheless, our corresponding effects were small and not statistically credible. By contrast, near-far quickness (NFQS and NFQO) requires repeated alternation between real near and far targets and therefore preserves more natural accommodative-vergence coupling; it also showed credible Pro-NoPro differences in our data, supporting near-far quickness as a more ecologically relevant assessment for many sport settings. Taken together, these considerations suggest that BAF/BVF may be better interpreted as clinical measures of visual efficiency rather than direct discriminators of sport expertise in heterogeneous athlete cohorts (Omar et al., 2017).

Previous studies have consistently shown that elite athletes exhibit superior perceptual-cognitive and VMF (e.g., (Alves et al., 2013; Burris et al., 2020). However, due to methodological limitations in many of those studies (such as small sample sizes or uncertain confounding age-related effects) it remains unclear whether these enhancements are primarily innate or shaped through training. The present study minimises age-related bias by focusing on a restricted age range (14-39 years), thereby strengthening the interpretation that VMF are primarily shaped by expertise rather than age by reducing, but not eliminating, age-related confounding. Furthermore, existing evidence supports the efficacy of structured visuomotor training in enhancing reaction time, anticipation, and decision-making (Romeas et al., 2016). Taken together, these findings suggest that VMF are not only markers of elite performance but may also be improved through targeted training, offering potential performance benefits for both developing and professional athletes.

Notable sex differences were observed in specific VMF, with female athletes scoring significantly better in DP and BAF, while male athletes performed better in CS and MOT. A trend of better PS, BVF, and NFQO scores in female athletes was also noted, although these differences were not statistically confirmed. These results partially align with study by Burris et al. (2020), who found superior PS and eye-hand coordination in females and better NFQS in males. However, no significant sex difference was observed in NFQS in the present study. The literature on sex differences in DP remains inconclusive, with some studies suggesting superior DP in females under specific conditions, such as in elderly populations (Ruhina & Sridevi, November 3, 2021), while others report no significant differences (Singh, 2018). Similarly, research on BAF is limited, with only one study comparing BAF between sexes, which found no significant differences in a sample of university students (Chikuse et al., December 3, 2022). Given the larger sample size in the present study, the observed sex differences in DP and BAF provide novel insights into sex-based visual performance.

In contrast, male athletes demonstrated superior CS, aligning with Oen et al. (1994), who reported better CS in males in a large sample study. However, this finding contradicts more recent studies that did not identify significant sex differences in CS (Burris et al., 2020), suggesting that CS may be influenced by multiple factors, including methodology and environmental conditions. The present findings on MOT are consistent with previous research indicating that males tend to perform better in multiple object tracking tasks (Jin et al., 2023). These findings highlight an important aspect of sports performance, suggesting that biological differences may contribute to how athletes develop and refine cognitive and VMF skills. These sex-specific patterns may warrant differentiated training strategies that leverage the distinct strengths observed in male and female athletes. Further research should explore whether training interventions can address these differences and optimise performance in both sexes. Notably, given the unequal distribution of sport types across sexes (Table 1), some observed sex differences may partly reflect sport-type composition.

Differences between strategic and interceptive sport athletes suggest that sport type imposes distinct cognitive demands. Athletes in strategic sports demonstrated significantly better MOT performance, which can be attributed to the need to track multiple players and anticipate movement patterns. This is in line with studies suggesting that strategic sport athletes exhibit superior working memory and cognitive flexibility compared to interceptive sport athletes (Liew et al., 2022; Pineda et al., 2022; Soltani & Morice, 2023; Yu & Liu, 2020). However, no other study has directly compared MOT performance between athletes from strategic and interceptive sports, highlighting the need for further investigation. The present findings differ from those of Burris et al. (2020), who reported significantly better VC, CS, NFQS, and RT in interceptive athletes and better PS in strategic athletes. This discrepancy may be attributed to differences in how groups were formed for analysis. Burris et al. (2020) included younger middle-school athletes, while the present study excluded participants younger than 14 years of age to minimise age-related biases. Given that VC is a fundamental visual ability primarily determined by anatomical factors rather than training, the lack of significant differences between Pro and NoPro athletes in this function supports the validity of this approach. However, sample size limitations, particularly in the interceptive sports group, must also be considered. Broader studies should also examine VMF in static sports and across athlete development stages to assess generalisability and training responsiveness.

Our findings further emphasise the complex interplay between VMF and sports training. Because optometrist interviews indicated that participants did not engage in structured visuomotor (sports vision) training, our findings suggest that Pro-NoPro differences in VMF are not solely innate but may reflect cumulative sport exposure; however, causality cannot be inferred. This is consistent with studies demonstrating that intensive training enhances action prediction abilities and perceptual-cognitive skills in athletes (Makris & Urgesi, 2015). The analysis of interdependencies among VMF revealed additional insights. VC and CS were closely related, confirming previous findings. BAF and BVF were significantly correlated, though prior research has reported mixed results, with some studies showing a simultaneous increase (Munsamy et al., 2020) or decrease (Padavettan et al., 2021) in both parameters following visual engagement, while others found divergent effects such as the work of Janagi et al. (2022) who found increase in BAF but decrease in BVF after 1 hour of mobile gaming. These findings suggest that BAF and BVF may be influenced by short-term activities, and optimising pre-competition warm-ups to enhance these parameters could improve athlete performance, consistent with reports that brief oculomotor and coordination exercises can acutely improve sensorimotor readiness in athletes (Matsuura et al., 2024). Future research should investigate whether such short-term activities modulate BVF/BAF and whether these effects translate into competitive contexts. Additionally, a strong correlation was observed between MOT and PS, which is likely due to the shared demand for tracking and processing multiple elements within a visual scene. In applied settings, the observed BAF-BVF coupling suggests that brief pre-competition vergence/accommodation drills could be evaluated as a means to acutely optimise visual efficiency, although this requires prospective testing.

The use of Self-Organising Maps (SOM) for data visualisation further supported these findings, identifying DP and RT as distinct from other VMF. This suggests that these abilities may be governed by unique neurophysiological mechanisms, which could have implications for sports vision training and clinical diagnostics. Further research is needed to explore these relationships and to determine whether targeted interventions can enhance these specialised VMF.

This study has certain limitations, and its results should be interpreted with caution. The retrospective, cross-sectional design precludes causal inference, while standardised data collection enhances reliability but not causality. Subgroup sizes were uneven (notably within NoPro and Interceptive strata), and the NoPro category pooled recreational, school-level, and collegiate athletes; these features may attenuate contrasts and reduce precision in subgroup estimates. Despite standardised intake forms and optometrist-validated interviews, minor misclassification of competitive status or sport type cannot be entirely ruled out. The age restriction (14-39 years) limits generalisability to younger and older athletes. Within this restricted range, we did not adjust for age, so residual age-related confounding remains possible. Training exposure (e.g., weekly volume, years in sport, and any prior prior vision training/therapy) was not captured in a standardised form and was therefore not modelled, which may leave residual confounding by unmeasured exposure. We also did not link VMF parameters to sport performance metrics (e.g., game statistics), which limits inferences about direct competitive impact. The study does not assess the effect of targeted VMF training, which future research should explore to establish the practical relevance of these findings for performance optimisation. Despite these limitations, this remains one of the most extensive studies on VMF in athletes, providing valuable insights into the influence of expertise, sex, and sport type, that can inform evidence-based training strategies. Finally, absolute differences in VMF parameters (e.g., reaction time in milliseconds) were not reported, as the Bayesian models operated on standardised and transformed scales. Future studies should include reporting in raw units to facilitate interpretation of practical effect sizes.

Beyond these limitations, this study helps situate single-task differences within a broader visuomotor profile. By combining a validated digital battery with clinic-based optometry in the same athletes, we were able to show convergent advantages for professionals on domains that are already considered important for sport (multiple object tracking, perception span, reaction time, and near-far quickness). These are also the domains that are most often reported as trainable or linked to on-field decision-making in prior sport-vision work (Murray et al., 2021; Romeas et al., 2016). Placing all ten domains in one multivariate model and visualising them on a self-organising map therefore provides clinicians and performance staff with a clearer rationale for which tests to prioritise in screening and monitoring.

From an applied sports vision perspective, the expertise-linked differences observed here are also the most practically actionable. Near-far quickness (NFQS and NFQO) maps onto the ability to shift visual focus efficiently between distant cues and near-space actions, which is common in many open-skill sports. MOT and PS reflect the capacity to distribute attention and extract task-relevant information from complex, dynamic scenes (e.g., multiple players, trajectories, and spatial relations), while RT captures time-critical response initiation. DP may be particularly relevant for interceptive actions that require precise spatial judgments for timing and contact. In contrast, VC and CS showed minimal differences by competitive level, supporting their primary role in identifying clinical visual deficits rather than differentiating sport expertise. Accordingly, when the goal is performance-oriented screening or monitoring, prioritising NFQS or NFQO, MOT, PS, and RT may provide the greatest practical value, whereas VC/CS may be most informative for clinical vision care and correction.

This study represents one of the most extensive investigations on VMF in athletes, showing that professional athletes outperform non-professionals on the visuomotor functions that place the greatest demand on dynamic attention, tracking, and rapid visual-motor responding, while basic visual clarity and contrast sensitivity remain similar across groups. Sex and sport-type differences further indicate that visuomotor demands are not uniform across athletes and should be profiled rather than assumed. Taken together with the multivariate analyses, these findings support the practical use of combined digital and optometric testing to guide sport-specific assessment and training.

Future studies should add standardised training exposure, longitudinal follow-up, and sport performance metrics to separate selection effects from training-related adaptation and to confirm that improvement on these visual-motor tasks transfers to competitive outcomes. In the meantime, programs that emphasise multiple object tracking, perception span, reaction time, and near-far quickness, and that are aligned with sport-type demands (for example, strategic versus interceptive sports), appear to be the most promising targets. Individualised visuomotor profiling may also be useful for return-to-play decisions and for planning workload progression after visual or neurological compromise. Understanding these group differences can help refine training approaches to better target sport-specific perceptual and cognitive skills.

## Data Availability

The data supporting this study were provided by the sports vision and optometry centre under a data-use agreement and are not publicly available due to contractual and privacy restrictions imposed by the provider. De-identified summary outputs underlying the main findings are included in the article’s tables and figures. Additional de-identified aggregates can be shared by the corresponding author upon reasonable request and with permission from the data provider.

## References

[cit0001] Allen, S. V., & Hopkins, W. (2015). Age of peak competitive performance of elite athletes: A systematic review. *Sports Medicine*, *45*(10), 1431–1441. 10.1007/s40279-015-0354-3 26088954

[cit0002] Alves, H., Voss, M. W., Boot, W. R., Deslandes, A., Cossich, V., Salles, J. I., & Kramer, A. F. (2013). Perceptual-cognitive expertise in elite volleyball players. *Front Psychol*, 4, 36–36. 10.3389/fpsyg.2013.00036 23471100 PMC3590639

[cit0003] Bollano-Lazaridis, M., & Chandrinos, A. (2021). Optometric screening of elementary students in Greece. *Ophthalmology Research: An International Journal*, *14*(August), 42–49. 10.9734/or/2021/v14i430201

[cit0004] Burris, K., Liu, S., & Appelbaum, L. (2020). Visual-motor expertise in athletes: Insights from semiparametric modelling of 2317 athletes tested on the nike SPARQ sensory station. *Journal of Sports Sciences*, *38*(3), 320–329. 10.1080/02640414.2019.1698090 31782684

[cit0005] Chikuse, M., Mzumara, T., & Afonne, J. (2022). Establishing normative values for amplitude of accommodation and accommodative facility among university students in Malawi. *Ophthalmology Research: An International Journal*, *17*, 51–56. 10.9734/or/2022/v17i4372

[cit0006] Christenson, G., & Winkelstein, A. (1988). Visual skills of athletes versus nonathletes: Development of a sports vision testing battery. *Journal of the American Optometric Association*, *59*(9), 666–675. 3183281

[cit0007] Clark, J., Betz, B., Borders, L., Kuehn-Himmler, A., Hasselfeld, K., & Divine, J. (2020). Vision training and reaction training for improving performance and reducing injury risk in athletes. *Journal of Sports and Performance Vision*, *2*, 8–16 10.22374/jspv.v2i1.44

[cit0008] Covassin, T., & Elbin, R. (2010). The cognitive effects and decrements following concussion. *Open Access Journal of Sports Medicine*, *1*, 55–61. 10.2147/OAJSM.S6919 24198543 PMC3781855

[cit0009] Erickson, G., Citek, K., Cove, M., Wilczek, J., Linster, C., Bjarnason, B., & Langemo, N. (2011). Reliability of a computer-based system for measuring visual performance skills. *Optometry*, *82*(9), 528–542. 10.1016/j.optm.2011.01.012 21705283

[cit0010] Gelman, A., & Rubin, D. (1992). Inference from iterative simulation using multiple sequences. *Statistical Science*, *7*(4), 457–472. 10.1214/ss/1177011136

[cit0011] Haynes, H. (1979). The distance rock test - a preliminary report. *Journal of the American Optometric Association*, *50*(6), 707–713. 479497

[cit0012] Hoff, P. D. (2007). Extending the rank likelihood for semiparametric copula estimation. *The Annals of Applied Statistics*, *1*(1), 265–283. 10.1214/07-AOAS107

[cit0013] Hülsdünker, T., Strüder, H., & Mierau, A. (2018). The athletes’ visuomotor system-cortical processes contributing to faster visuomotor reactions. *European Journal of Sport Science*, *18*, 955–964. 10.1080/17461391.2018.1468484 29738678

[cit0014] Janagi, S., Ikram, S., & Puri, S. (2022). Accommodative facility and vergence facility after mobile gaming. *BOHR International Journal of Current Research in Optometry and Ophthalmology*, *1*(1), 104–106. 10.54646/BIJCROO.025

[cit0015] Jin, P., Zhao, Z., & Zhu, X. (2023). The relationship between sport types, sex and visual attention as assessed in a multiple object tracking task. *Frontiers in Psychology*, *14*, 1–7. 10.3389/fpsyg.2023.1099254 PMC9996032 36910748

[cit0016] Kirby, E., Jones, K., Campbell, N., Fickling, S., & D’Arcy, R. (2025). Objective neurophysiological measures of cognitive performance in elite ice hockey players. *Open Access Journal of Sports Medicine*, *16*, 15–24. 10.2147/OAJSM.S494589 39877760 PMC11774107

[cit0017] Kramer, M., Teel, E., Wasserman, E., & Mihalik, J. (2021). Effect of protective helmets on vision and sensory performance in healthy men. *Athletic Training & Sports Health Care*, *13*(3), 130–135. 10.3928/19425864-20200206-01

[cit0018] Legarreta, A. D., Mummareddy, N., Yengo-Kahn, A. M., & Zuckerman, S. L. (2019). On-field assessment of concussion: clinical utility of the King-Devick test. *Open Access Journal of Sports Medicine*, *10*, 115–121. 10.2147/OAJSM.S171815 31686924 PMC6709031

[cit0019] Liew, E., Chen, W., Chiang, C., Song, T., & Wu, S. (2022). Multiple Object Tracking Ability of Athletes in Different Interceptive Sports. *大專體育學刊.*, *24*(4), 530–545. 10.5297/ser.202212_24(4).0007

[cit0020] Makris, S., & Urgesi, C. (2015). Neural underpinnings of superior action prediction abilities in soccer players. *Social Cognitive and Affective Neuroscience*, *10*(3), 342–351. 10.1093/SCAN/NSU052 24771282 PMC4350476

[cit0021] Mann, D., Williams, A., Ward, P., & Janelle, C. (2007). Perceptual-cognitive expertise in sport: A meta-analysis. *Journal of Sport and Exercise Psychology*, *29*(4), 457–478. 10.1123/jsep.29.4.457 17968048

[cit0022] Matsuura, Y., Sakairi, Y., Sato, H., & Takiura, K. (2024). Do combined oculomotor and bimanual coordination exercises instantly stabilize balance in athletes? *Open Access Journal of Sports Medicine*, *15*, 77–89. 10.2147/OAJSM.S472125 39049901 PMC11268565

[cit0023] Munsamy, A., Paruk, H., Gopichunder, B., Luggya, A., Majola, T., & Khulu, S. (2020). The effect of gaming on accommodative and vergence facilities after exposure to virtual reality head-mounted display. *Journal of Optometry*, *13*(3), 163–170. 10.1016/J.OPTOM.2020.02.004 32234359 PMC7301196

[cit0024] Murray, N., Lawton, J., Rider, P., Harris, N., & Hunfalvay, M. (2021). Oculomotor behavior predict professional cricket batting and bowling performance. *Frontiers in Human Neuroscience*, *15*(December), 1–8. 10.3389/fnhum.2021.768585 PMC8739967 35002657

[cit0025] Niechwiej-Szwedo, E., Meier, K., Christian, L., Niechwiej‐Szwedo, E., Nouredanesh, M., Tung, J., Bryden, P., & Giaschi, D. (2020). Concurrent maturation of visuomotor skills and motion perception in typically-developing children and adolescents. *Developmental Psychobiology*, *62*(3), 353–367. 10.1002/dev.21931 31621075

[cit0026] Oen, F., Lim, T., & Chung, M. (1994). Contrast sensitivity in a large adult population. *Annals of the Academy of Medicine, Singapore*, *23*(3), 322–326. 7944242

[cit0027] Oh, A., Chen, T., Shariati, M., Jehangir, N., Hwang, T., & Liao, Y. (2018). A simple saccadic Reading test to assess ocular motor function in cerebellar ataxia. *PLoS One*, *13*(11), 1–18. 10.1371/journal.pone.0203924 PMC6221255 30403759

[cit0028] Omar, R., Kuan, Y., Zuhairi, N., Manan, F., & Knight, V. (2017). Visual efficiency among teenaged athletes and non-athletes. *International Journal of Ophthalmology*, *10*(9), 1460. 10.18240/IJO.2017.09.20 28944208 PMC5596234

[cit0029] Padavettan, C., Nishanth, S., Vidhyalakshmi, S., Madhivanan, N., & Madhivanan, N. (2021). Changes in vergence and accommodation parameters after smartphone use in healthy adults. *Indian Journal of Ophthalmology*, *69*(6), 1487–1490. 10.4103/IJO.IJO_2956_20 34011725 PMC8302311

[cit0030] Pineda, R., Krampe, R., Vanlandewijck, Y., & Van Biesen, D. (2022). Cognitive–motor multitasking in athletes with and without intellectual impairment. *Scandinavian Journal of Medicine and Science in Sports*, *32*(2), 424–434. 10.1111/sms.14088 34706114

[cit0031] Poltavski, D., & Biberdorf, D. (2015). The role of visual perception measures used in sports vision programmes in predicting actual game performance in division I collegiate hockey players. *Journal of Sports Sciences*, *33*(6), 597–608. 10.1080/02640414.2014.951952 25142869

[cit0032] Romeas, T., Guldner, A., & Faubert, J. (2016). 3D-Multiple object tracking training task improves passing decision-making accuracy in soccer players. *Psychology of Sport and Exercise*, *22*, 1–9. 10.1016/j.psychsport.2015.06.002

[cit0033] Roy, V. (2020). Convergence diagnostics for Markov chain Monte Carlo. *Annual Review of Statistics and Its Application*, *7*(1), 387–412. 10.1146/annurev-statistics-031219-041300

[cit0034] Ruhina, A., & Sridevi, G. (2021). An evaluation of visual perception visual memory cognitive functions and emotional status among genders in elderly subjects. *J Pharm Res Int*, 330–336. 10.9734/jpri/2021/v33i47b33132

[cit0035] Sarvestan, J., Svoboda, Z., Baeyens, J., & Serrien, B. (2023). Whole body coordination patterning in volleyball spikes under various task constraints: Exploratory cluster analysis based on self-organising maps. *Sports Biomechanics / International Society of Biomechanics in Sports*, *22*(9), 1153–1167. 10.1080/14763141.2020.1788132 32744139

[cit0036] Singh, H. (2018). Comparative study of depth perception among the female players of handball. *International Journal of Yogic, Human Movement and Sports Sciences*, *3*(1), 954–955. www.theyogicjournal.com

[cit0037] Soltani, P., & Morice, A. (2023). A multi-scale analysis of basketball throw in virtual reality for tracking perceptual-motor expertise. *Scandinavian Journal of Medicine and Science in Sports*, *33*(2), 178–188. 10.1111/sms.14250 36315055 PMC10100508

[cit0038] Stalin, A., & Dalton, K. (2020). Relationship of contrast sensitivity measured using quick contrast sensitivity function with other visual functions in a low vision population. *Investigative Ophthalmology and Visual Science (Philadelphia, PA)*, *61*(6), 21. 10.1167/IOVS.61.6.21 PMC7415278 32516407

[cit0039] Voss, M., Kramer, A., Basak, C., Prakash, R., & Roberts, B. (2010). Are expert athletes ‘expert’ in the cognitive laboratory? A meta-analytic review of cognition and sport expertise. *Applied Cognitive Psychology*, *24*(6), 812–826. 10.1002/acp.1588

[cit0040] Wang, L., Krasich, K., Bel-Bahar, T., Hughes, L., Mitroff, S., & Appelbaum, L. (2015). Mapping the structure of perceptual and visual-motor abilities in healthy young adults. *Acta Psychologica (Amsterdam)*, *157*, 74–84. 10.1016/j.actpsy.2015.02.005 25747573

[cit0041] Wick, B., Gall, R., & Yothers, T. (2002). Clinical testing of accommodative facility: Part III. Masked assessment of the relation between visual symptoms and binocular test results in school children and adults. *Optometry*, *73*(3), 173–181. 12365701

[cit0042] Yu, M., & Liu, Y. (2020). Differences in executive function of the attention network between athletes from interceptive and strategic sports. Published online. 10.1080/00222895.2020.1790486 32654658

[cit0043] Yu, C., Cheng, M., An, X., Chueh, T., Wu, J., Wang, K., & Hung, T. (2025). The effect of EEG neurofeedback training on sport performance: A systematic review and meta-analysis. *Scandinavian Journal of Medicine and Science in Sports*, *35*(5), e70055. 10.1111/sms.70055 40270441 PMC12019780

[cit0044] Zwierko, T., Puchalska-Niedbał, L., Krzepota, J., Markiewicz, M., Woźniak, J., & Lubiński, W. (2015). The effects of sports vision training on binocular vision function in female university athletes. *Journal of Human Kinetics*, *49*, 287–296. 10.1515/hukin-2015-0131 26925183 PMC4723179

